# Microbial Population and Physicochemical Properties of Miang Fermented in Bamboo Tubes by the Luar Ethnic Group in Lao PDR

**DOI:** 10.3390/foods13132109

**Published:** 2024-07-02

**Authors:** Somsay Phovisay, Aliyu Dantani Abdullahi, Nang Nwet Noon Kham, Kridsada Unban, Kalidas Shetty, Chartchai Khanongnuch

**Affiliations:** 1 Multidisciplinary School, Chiang Mai University, Chiang Mai 50200, Thailand; somsay2009@hotmail.com (S.P.); abdullahialiyud.ada@gmail.com (A.D.A.); nwenoonkham@gmail.com (N.N.N.K.); 2 Division of Food Science and Technology, Faculty of Agro-Industry, Chiang Mai University, Chiang Mai 50100, Thailand; 3 Research Center for Multidisciplinary Approaches to Miang, Multidisciplinary Research Institute (MDRI), Chiang Mai University, Chiang Mai 50200, Thailand; 4 Global Institute of Food Security and International Agriculture (GIFSIA), Department of Plant Sciences, North Dakota State University, Fargo, ND 58108, USA; kalidas.shetty@ndsu.edu; 5 Department of Biology, Faculty of Science, Chiang Mai University, Huay Kaew Rd., Chiang Mai 50200, Thailand; 6 Research Center of Microbial Diversity and Sustainable Utilization, Chiang Mai University, Huay Kaew Rd., Chiang Mai 50200, Thailand

**Keywords:** fermented tea, microbial community, nutritional composition, bioactive compounds, antioxidants

## Abstract

Miang is a traditional fermented food made from Assam tea leaves and consumed as a snack. This study investigated the underground Miang fermentation process practiced by the Luar ethnic group in Laos, specifically examining the nutritional composition and microbial dynamics. Lactic acid bacteria and yeast were dominant in the fermentation process, reaching 8.43 and 8.50 log CFU/g after one week before gradually declining, while the coliform bacterial count was at 5.31 log CFU/g in the initial week but became undetectable in the later stages of fermentation. Next-generation sequencing identified *Firmicutes* (75.02%) and *Proteobacteria* (23.51%) as the primary phyla. Bacterial genera included *Lactobacillus* (73.36%) and *Acetobacter* (21.06%), with fungi mainly represented by *Pichia* (85.52%) and *Candida* (13.45%). Fundamental microbes such as *Lactobacillus* and *Acetobacter* were predominantly present, alongside *Pichia* and *Candida*, in the fungal communities. Microbial activities played a crucial role in generating essential enzymes for Miang’s transformation. The nutritional transformation appears to be complete at 5 weeks of fermentation. The moisture content in the final products was approximately 74% and correlated with a change in nitrogen-free extract (NFE) and crude fiber. The fat content showed a slight increase from 1.3% to 2.52%, but protein content slightly declined from 17.21% to 16.05%, whereas ash content did not change significantly. Key polysaccharide-degrading enzymes, particularly pectinase and β-mannanase, were revealed and peaked at 48.32 and 25.32 U/g Miang, respectively. The total polyphenols increased from 103.54 mg/g dry Miang to 144.19–155.52 mg/g during fermentation. The lowered IC_50_ value indicated an increase in antioxidant activity. A fermentation period of at least 3 weeks proved to be optimal for enhancing antioxidant properties and bioactive compounds, and mitigating the risk of coliform bacteria.

## 1. Introduction

The fermented Assam tea leaves, known as Miang, hold a prominent place in the culinary traditions of northern Thailand and north-western Lao PDR. Miang stands apart as an ethnic delicacy; unlike black tea, which is typically consumed as a beverage, Miang is chewed or consumed as a snack. Variations of Miang and analogous fermented tea products have been identified across diverse regions under different names, such as Suancha or Yancha in China, Laphet in Myanmar, and also Goishi-cha and Awaban-cha in Japan [1,2,3]. The fermented tea leaves underwent transformations in chemical properties, organic acid qualities, flavor, and taste due to microbial activities, which also generated bioactive metabolites with health benefits [4,5]. The tea leaves, *Camellia sinensis* var. assamica, are currently fermented by native tribes, continuing long-standing traditions in Assam and Arunachal Pradesh in northeast India, South China, Myanmar, northwestern Laos, and northern Thailand. These products are used as a beverage, chewing tea, or food ingredient. In particular, Laos and Thailand have been known to ferment leaves of the *Assamica* variety into Miang “fermented Assam tea” products by incubating them inside bamboo tubes, in bamboo baskets, and burying them in pots [6]. Tea gardens in Lao PDR are divided into three main categories, including wild forest tea, cultivated forest tea (locally known as “ancient tea”), and commercially cultivated tea (introduced varieties). A study conducted across nine northern provinces in 2010–2011 identified approximately 8500 households involved in tea cultivation. The current tea-producing area is approximately 12,000 ha, of which 72% is wild forest tea, 24% commercially cultivated tea, and 4% ancient tea (National Agriculture and Forestry Research Institute, 2016). Tea products in Laos are currently mostly processed into beverages due to the recent trend responding to market demand and include green tea, Mao-cha (rough tea used to produce a fermented tea called “Pu-erh tea”), and other teas such as white tea, black tea, yellow tea, red tea, and oolong tea. However, the traditional product of fermented Assam tea, “Miang” (eating tea), has been restricted to a minority community in the northwest of Lao PDR, which once belonged to the ancient Lanna Kingdom and has a long history of Miang cultivation and consumption for over hundreds of years until now [7]. Miang had a crucial role in the sociocultural traditions and commerce in the ancient Lanna Kingdom, which was composed of modern-day north Thailand, northeastern Myanmar, and the northwestern part of Laos [8]. Miang processing involves a traditional method of transforming Miang leaves into a local delicacy through biotransformation involving natural microflora. These microorganisms work to enhance the flavor profile, imbuing the steamed-fresh Miang leaves with an improved and distinctive sour taste [9,10,11,12]. Likewise, Miang leaves contain abundant bioactive compounds with health benefits, including antimicrobial and antioxidant activities [13,14]. Previous studies reported that lactic acid bacteria (LAB), such as *L. pentosus* and *L. plantarum* [15,16], and yeast *Candida ethanolica* and *Pichia manshurica* [17], were the dominant microbial population during the fermentation process. Moreover, LAB isolated from Miang exhibited probiotic potential and the production of extracellular bacteriocins, suggesting their potential for conferring health benefits [18,19] Similar to other fermented foods, the properties of fermented tea are shaped by a combination of factors, including the microbial community, geographical conditions, regional varieties, and the specific fermentation process. These factors collectively influence the ultimate qualities of the final product [10,20]. The Luar ethnic group in Laos produces Miang through a natural fermentation process using locally sourced raw materials. This method incorporates indigenous knowledge to create a traditional fermented tea that holds cultural significance. Miang leaves are first steamed, then carefully packed into bamboo tubes and buried underground for fermentation, a process that spans from one week to several months [21]. However, there is currently a lack of scientific studies and publications detailing the mechanisms of Miang fermentation in the underground incubation process. No research has yet explored the intricacies of the fermentation process in this context, particularly whether the conditions of incubation temperature play a significant role in influencing the species diversity of the microbial population. This results in noticeable variations along the temperature gradient throughout the fermentation process [20]. Moreover, it is likely that biotransformation occurring during the Miang fermentation process generates microbial activities that help suppress soil pathogens in the subterranean fermentation environment. However, scientific research on comprehending the nutritional properties and microbial community of Miang remains limited. Consequently, this research endeavors to provide a deeper understanding of the underground Miang fermentation process. Therefore, this study aimed to investigate the changes in nutritional properties, bioactive compounds, and the microbial community throughout the various stages of fermentation.

## 2. Materials and Methods

### 2.1. Raw Material and Chemicals

Young leaves of *Camellia sinensis* var. *assamica*, commonly referred to as Miang leaves by local people, were harvested from the tea plantation of the Luar ethnic group located in the Saysathan district of Xayabouty province, Lao PDR. The reagents 3,5-dinitrosalicylic acid, 1,1-diphenyl-2-picrylhydrazyl (DPPH), gallic acid, quercetin, and PVPP were procured from Sigma-Aldrich (St. Louis, MO, USA). De Man, Rogosa, and Sharpe, Sabouraud dextrose agar, and MacConkey agar were purchased HiMedia (Mumbai, India). Chloramphenicol was obtained from Bio Basic Inc. (Markham, ON, Canada). The substrates used for enzyme assay were carboxymethyl cellulose (Nacalai Tesque, Kyoto, Japan), beech wood xylan (Megazyme, Bray, Ireland), pectin (Sigma-Aldrich, St. Louis, MO, USA), soluble starch (Fisher Scientific, Loughborough, UK), and locust bean gum (Sigma-Aldrich, St. Louis, MO, USA). Folin–Ciocalteu phenol reagent was purchased from LOBA Chemie (Mumbai, India). Genomic DNA extraction was carried out using the DNeasy PowerSoil kit from Qiagen (Hilden, Germany). Analytical reagent (AR)-grade chemicals, such as sodium chloride, sodium hydroxide, sodium phosphate, sodium carbonate, aluminum nitrate, potassium acetate, petroleum ether, methanol, acetone, and sulfuric acid, were also used.

### 2.2. Sample Preparation

**Figure 1:**
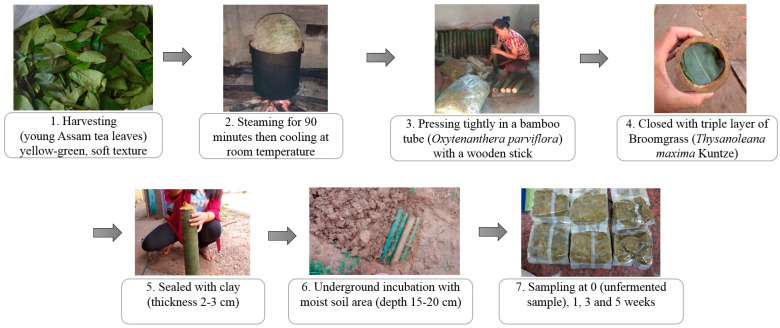
Flowchart diagram of Miang processing by the Luar ethnic group.

Miang fermentation was initiated following a native method of the Luar ethnic group (Figure 1) at the local Miang production site (N19.405888″, E101.387887″) in Saysathan district, Xaiyaboury province, Lao PDR. Firstly, young Miang leaves were harvested and wrapped into bundles then steamed for 90 min. The steamed leaves were cooled at room temperature overnight. Subsequently, bamboo tubes (*Dendrocalamus hamiltonii)* with an internode were prepared and cleaned to be the fermenting container. The steamed leaves were pressed tightly inside bamboo tubes and then closed by a triple layer of broom grass (*Thysanoleana maxima* Kuntze), and finally sealed by clay (thickness 2–3 cm). The Miang tubes were transferred to the soil cavity and completely filled with soil to process and allow underground fermentation for 5 weeks. Miang samples were harvested from each batch at specific time intervals of 0, 1, 3, and 5 weeks. Each sample was collected aseptically in a plastic bag, kept in an ice box, and subsequently stored in a chilling room at 4 °C at the Faculty of Agro-Industry, Chiang Mai University, Thailand, until further analysis.

### 2.3. Enumeration of Microorganisms in the Miang Sample

Microbial populations were enumerated using selective agar methods. Briefly, a Miang sample weighing 25 g was blended with 225 mL of sterile 0.85% (*w*/*v*) sodium chloride solution and mixed thoroughly using a stomacher (Masticator Homogenizator, IUL Instruments, Barcelona, Spain) for 10 min. Subsequent decimal dilutions were prepared using the same diluent, followed by culturing on specific agar media. Enumeration of the viable microorganisms included the total plate count on nutrient agar, lactic acid bacteria on De Man, Rogosa, and Sharpe (MRS) agar containing 0.015% (*w*/*v*) bromocresol purple, yeast on Sabouraud dextrose agar supplemented with chloramphenicol (100 mg/L), and coliform bacteria on MacConkey agar. Additionally, the residual diluted solutions were further incubated in a water bath at 80 °C for 12 min, and each dilution was spread onto nutrient agar to determine the viable cell count of endospore-forming *Bacillus* spp. [22].

### 2.4. Identification of Microbial Diversity of Miang Product by Next-Generation Sequencing

The cell pellets were collected randomly from at least five different portions of the final Miang product after 5 weeks of fermentation. The genomic DNA was then extracted from these pellets using the DNeasy PowerSoil kit (Qiagen, Hilden, Germany) following the manufacturer’s instructions. Electrophoresis analysis was used to check genomic DNA on 1% agarose gel. The purity and concentration were estimated using a Nanodrop-2000 spectrophotometer (Thermo Fisher Scientific, Inc., Waltham, MA, USA). Five of the extracted genomic DNA were combined and transferred to one tube (size 1.5 mL), which was used as the final genomic DNA template for PCR. Amplicon-based next-generation sequencing was used to determine the bacterial community present in Miang. The variation (V3-V4) region of the bacterial 16S rRNA gene was amplified using the primers 341-F/806-R (5′-CCT AYG GGR BGC ASC AG-3′ and 5′-GGA CTA CNN GGG TAT CTA AT-3′). For fungal communities present, the ITS rRNA gene was amplified with the forward primer ITS3 (5′-GCA TCG ATG AAG AAC GCA GC-3′) and the reverse primer ITS4 (5′-TCC TCC GCT TAT TGA TAT GC-3′). The sequenced libraries of bacterial 16S rRNA genes and fungal ITS rRNA genes were generated for high-throughput sequencing using TruSeq DNA PCR-Free Sample Preparation Kit (Illumina, San Diego, CA, USA). Sequencing data processing was conducted where paired-end reads were assigned to samples based on their unique barcodes. After truncating the barcode and primer sequences, FLASH (V1.2.11) was used to merge the reads into raw tags. Quality control was performed using FASTP software (version 0.20.1) to obtain high-quality clean tags. Vsearch software (version 2.4.3) was then employed to detect and remove chimeras. For denoising, the DADA2 module in QIIME2 was utilized, filtering out sequences with an abundance of less than 5 to generate the final ASVs (amplicon sequence variants) and feature table. Species annotation for each ASV was conducted using the Classify-sklearn module in QIIME2 software (version 2019.10) to obtain the species information of each ASV.

### 2.5. Chemical Properties of pH and Total Acid Titration

Each sample of 10 g of Miang product, taken at specific time intervals, was suspended in 100 mL of distilled water and well mixed by a stomacher for 10 min using a Masticator homogenizer. The supernatant of the homogenized sample was separated using centrifugation (Velocity 14R, Dynamica Scientific Ltd., Livingston, UK) at 8000 rpm and 4 °C. The pH value and total acid titration (AOAC, 2012) were determined by pH meter using an OHAUS starter 2100 pH meter (Pine Brook, NJ, USA) and NaOH (0.01 M), respectively.

### 2.6. Investigation of Polysaccharide-Degrading Enzymes in Miang Fermentation

Polysaccharide-degrading enzymes, cellulase, β-mannanase, xylanase, pectinase, and amylase, were quantified using the 3,5-dinitrosalicylic acid (DNS) method [23]. Briefly, the supernatant was collected from Miang suspension in sterile water. Then, the reaction mixture containing 0.125 mL of the proper dilution of the enzyme from the supernatant and 0.125 mL of 0.5% (*w*/*v*) locust bean gum (LBG) in 0.1 M sodium phosphate buffer (pH 5.0) was used as a substrate, incubated in a water bath (digital water bath LWB-122D, Labtech, Namyangju, Republic of Korea) at 37 °C for 10 min. The enzyme reaction was terminated by adding 0.25 mL of DNS, boiling for 10 min, and adding 2 mL of distilled water, and the absorbance was measured by a spectrophotometer at 540 nm (Drawell Scientific Instrument, Chongqing, China). The β-mannanase activity unit (U) was defined as the capability of the enzyme to liberate 1 μmole of reducing sugar from the substrate solution per minute [24]. Similarly, the activities of cellulase, xylanase, pectinase, and amylase were determined via the reducing-sugar-forming capacity analyzed by the DNS method, following the same procedure. Specific substrates were used for each enzyme, including 0.5% (*w*/*v*) carboxymethyl cellulose (CMC) for cellulase, beech wood xylan for xylanase, pectin for pectinase, and soluble starch for amylase. These substrates were prepared in 0.1 M sodium phosphate buffer pH 5. The enzyme reactions were performed under the same conditions as previously described.

### 2.7. Proximate Analysis

The moisture, carbohydrate, fat, protein, fiber, and ash components of Miang samples were determined using the AOAC (2012) method. In brief, for moisture content evaluation, 3 g of each sample was placed in a moisture can and subjected to vacuum drying at 50 °C using a Binder VD 155 oven (GmbH, Tuttlingen, Germany) until a constant weight was achieved. After cooling, the sample was transferred to a desiccator and weighed to calculate moisture content. Crude fat determination was automated using a Soxhlet^TM^ 8000 extraction unit (Foss, Hoganas, Sweden) with a manual method. Petroleum ether was employed for extraction, and the resulting fat content was calculated as crude fat percentage. For protein assessment, nitrogen content was analyzed using a combustion analyzer FP-528 (Leco, St. Joseph, MI, USA). The extracted nitrogen content was converted to protein using a conversion factor of 4.3 to calculate crude protein content [25]. Crude fiber analysis was conducted using the semi-automatic Fibertec^TM^ 8000 (Foss, Hoganas, Sweden) system. The process involved sequential boiling with 1.25% (*w*/*v*) sulfuric acid and 1.25% (*w*/*v*) sodium hydroxide solutions to extract fiber from the sample. For ash content determination, a Carbolite furnace CWF1100 (Carbolite, Parsons Lane, Dartford, UK) was employed. A dried sample of 3 g was placed in a crucible and completely incinerated at 550 °C for 8 h. The residue was allowed to cool within a desiccator and then weighed to calculate ash content. The percentage of nitrogen-free extract (NFE) or carbohydrate was calculated using the following equation:

Nitrogen-free extract (%) = 100 − (% of crude fiber + crude fat + crude protein + ash)(1)

### 2.8. Extraction of Bioactive Compounds

Miang samples were dried using a vacuum drier (Binder VD 155 oven, GmbH, Tuttlingen, Germany) at 50 °C for 24 h and subsequently homogenized using a blender (Philips HR2051/00, Bangkok, Thailand). For extraction, 5 g samples of the homogenized Miang were treated with 100 mL of 80% (*v*/*v*) acetone. The extraction solution underwent incubation on an incubator shaker (LabTech LSI-3016R, Namyangju, Republic of Korea) set at 30 °C for 1 h. Following this, the extracted solution was subjected to filtration using Whatman filter paper with a pore size of 20 μm. Subsequently, the solvent was removed via a vacuum at 40 °C using a rotary evaporator (Eyela N1001V, Tokyo, Japan) for 20 min. The resulting extract was then stored at −20 °C (Sanden freezer SCF-0365, Bangkok, Thailand) for further analysis.

Total polyphenol content (TP) was measured by Folin–Ciocalteu reagent according to the previously published method [26]. Briefly, 200 µL of the Miang extracted sample was added into a test tube containing 200 µL of 2 M Folin–Ciocalteu reagent and vortexed (Vortex genie 2, Scientific Industries, New York, NY, USA). After adding 1.8 mL of deionized water, the sample was incubated at room temperature for 3 min. Then, 400 µL of 10% (*w*/*v*) sodium carbonate was added and vortexed. The final volume was adjusted to 4 mL by deionized water and incubated again without light exposure at room temperature for 1 h. The absorbance of blue coloration was measured at 725 nm using a spectrophotometer (Drawell Scientific Instrument, Chongqing, China). Gallic acid was used as the standard and the results are expressed as milligrams of gallic acid equivalents (GAE) per gram of sample.

The total tannin (TA) was determined by a modification of the Folin–Ciocalteu reagent [27]. Tannin was separated from other phenols by using polyvinylpolypyrrolidone (PVPP). Briefly, 1 mL of the Miang extract sample was mixed with 1 mL of 10% (*w*/*v*) PVPP, vortexed (Vortex genie 2, Scientific Industries, New York, NY, USA), and kept at 4 °C for 15 min; then, the solution was centrifuged (Velocity 14R, Dynamica Scientific Ltd., Livingston, UK) at 3000 rpm for 10 min and the supernatant was collected. The total phenol content of the PVPP-precipitated supernatant was measured using the Folin–Ciocalteu reagent, and total tannin was estimated using the following formula:

TA = TP − TP_PVPP precipitation_ (2)

The total flavonoid content (TF) was determined by the aluminum chloride colorimetric method. Briefly, 100 µL of 10% (*w*/*v*) of aluminum nitrate and 100 µL of 1 M potassium acetate were mixed with 500 µL of sample. Then, 3.3 mL of 80% (*v*/*v*) methanol was added to the reaction mixture in a test tube and incubated in the dark at room temperature for 40 min. The absorbance of the reaction was measured by a spectrophotometer at 415 nm (Drawell Scientific Instrument, Chongqing, China). Then, the absorbance values were estimated to total flavonoid content compared with the quercetin equivalents standard curve [26].

The antioxidant activity of the Miang extracts was analyzed according to a previously described method with slight modifications [28]. Briefly, 4 mL of 0.15 mM DPPH in methanol solution 80% (*v*/*v*) was added to 1 mL of diluted extract sample (5, 2.5, 1.0, and 0.5 mg/mL) in methanol solution 80% (*v*/*v*) and mixed vigorously. After 30 min of incubation in the dark at room temperature, the absorbance was measured by a spectrophotometer at 517 nm (Drawell Scientific Instrument, Chongqing, China). The radical scavenging percentage was calculated by comparing against a blank using the following formula:

Inhibition (%) = (1 − (B/A)) × 100(3)

where A represents the absorbance of the mixture without the extract and B represents the absorbance of the mixture containing the extract from the samples, and this inhibition percentage was used to create a graphical representation of inhibition against concentration, with DPPH scavenging activities quantified by the IC_50_ value indicating the concentration at which inhibition reaches half of its maximum level.

### 2.9. The Physical Properties of Color and Hardness

The color characteristics of Miang leaves were measured by a Chroma meter CR-400 (Konica Minolta, Osaka, Japan) and reported in the CIELab color system (L* a* b*). Texture analysis was measured in terms of hardness using TPA mode by a texture analyzer (Stable Micro System Ltd., Godalming, Surrey, UK). Briefly, 10 g of the Miang sample was formed into a cylindrical shape by pressing the Miang sample lightly into a cylindrical block (height 10 mm and diameter 30 mm). Hardness was measured using a P/50 probe (50 mm diameter) with a pre-test speed of 1.00 mm/s, 1.00 mm/s testing speed, and 5.00 mm/s post-test speed, and compressed twice with a 60% value.

### 2.10. Statistical Analysis

The significance of the difference was analyzed using a one-way analysis of variance (ANOVA). Means comparison was used to describe the statistical significance of the differences by Duncan’s multiple range test (*p* < 0.05) with SPSS software, version 17.

## 3. Results and Discussion

### 3.1. Microbial Enumeration during Miang Fermentation

**Figure 2:**
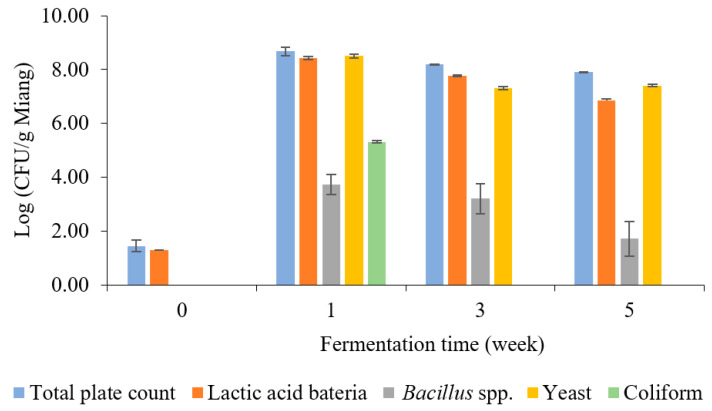
The microbial population during Miang fermentation in bamboo tubes with underground incubation.

The enumeration of microbial counts related to the Miang fermentation process was investigated periodically, as indicated in Figure 2. Lactic acid bacteria (LAB) and yeast were the predominant microbes during Miang fermentation. After streaming, the total plate count was 1.45 log CFU/g Miang in the unfermented sample. Then, the viable cell count increased sharply at 1 week, with a total plate count of 8.67 log CFU/g Miang, and then showed a steady decrease to 8.18 and 7.90 log CFU/g Miang at the end of fermentation. LAB increased sharply at 1 week to 8.43 log CFU/g Miang and then declined to 7.77 and 6.86 log CFU/g Miang. LAB such as *Lactoplantibacillus plantarum* and *Lactobacillus pentosus* in the Miang fermentation process were considered to provide a probiotic function as these microbes have been found widely in Miang fermentation [15,29,30]. Yeast was determined to be one of the major microbes during the Miang fermentation process, which increased to 8.50 log CFU/g Miang in a week and then gradually decreased to 7.31 and 7.40 log CFU/g Miang. Yeast was expected to play a significant role in metabolizing polysaccharide and enzyme activities, which encourage the formation of flavors and texture profiles of the final product, particularly *Candida ethanolica* and *Pichia manshurica*, which were previously reported as the major yeast species in fermented tea [17,31]. *Bacillus* spp. was 3.74 log CFU/g Miang at 1 week and declined steadily to 3.21 and 1.17 log CFU/g Miang. *Bacillus* spp. were expected to have important roles using their enzyme activity in the initial stage of fermentation [22,32].

Coliform bacteria were detected at 5.31 log CFU/g Miang in the first week, and this result highlights a hygiene problem in Miang due to the process being performed in the traditional manner, and Miang is mostly made by individual village communities. However, with the prolonged fermentation process, a decline in coliform bacteria was observed, and they finally became undetectable during the late fermentation (weeks 3–5). This phenomenon suggested the potential of antimicrobial activity linked to phenolic or phenolic-related compounds in tea, such as tannin and its derivatives, polyphenols, flavonoids, and catechins, which may have a role in the reduction of coliform bacteria [33]. In addition, a previous experiment using the agar diffusion method showed the capability of Miang extract to inhibit the growth of *Escherichia coli* and *Salmonella enterica* [19]. The inhibition mechanism potentially may be by cell disruption on the outer membrane, leading to cellular enzyme inactivation and membrane permeability changes that cause disruption of the plasma membrane leading to cell death [31]. Moreover, LAB isolated from fermented tea showed antibacterial potential against pathogenic bacteria [34] (*Shigella sonnei*, *Listeria monocytogenes*, *Streptococcus aureus*, and *Salmonella typhi*). As a result of this study, it could be suggested that the fermentation of Miang for at least 3 weeks would improve pathogen safety by avoiding the risk of coliform bacteria in Miang fermentation in bamboo tubes with underground incubation by the Luar ethnic group in Lao PDR.

### 3.2. Microbial Diversity during Miang Fermentation Using Next-Generation Sequencing Analysis

**Figure 3:**
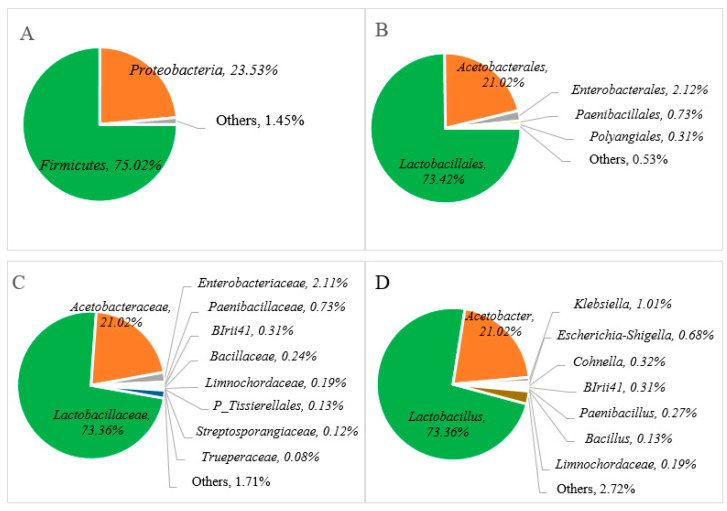
Relative abundance of bacterial communities at the phylum (**A**)**,** order (**B**)**,** family (**C**), and genus (**D**) levels of Miang fermented in bamboo tubes with underground incubation.

In order to examine the microbial diversity present during Miang fermentation, the hypervariable V3-V4 region of the 16S rRNA gene and ITS region were amplified by using the primers 341-F/806-R and ITS3F/ITS4R. A total of 327,273 sequencing reads were obtained from single bacterial and fungal PCR amplicons. The cleaned data of 240,899 sequencing reads of whole sequences involved about 103.781 sequencing reads (bacterial) and 137.118 sequencing reads (fungal), with an average read length of approximately 430 bp (bacterial) and 280 bp (fungal), which indicated taxonomic classification using relative abundance at the phylum, order, family, and genus levels to examine the microbial diversity of the Miang product. The relative abundance of the bacterial communities is presented in Figure 3, and it was found that two major phyla were allocated to the phylum of *Firmicutes* (75.02%), *Proteobacteria* (23.51%), and others (1.40%) in Figure 3A. At the order level, *Lactobacillales* (73.42%), *Acetobacterales* (21.02%), *Enterobacterales* (2.12%), *Paenibacillales* (0.73%), *Polyangiales* (0.31%), and others (0.53%) (Figure 3B) were confirmed. In terms of bacterial diversity, *Lactobacillaceae* was predominant family (73.36%) followed by families of *Acetobacteraceae* (21.02%), *Enterobacteriaceae* (2.11%), *Paenibacillaceae* (0.73%), *BIrii41* (0.31%), *Paenibacillus* (0.27%), *Limnochordaceae* (0.19%), *Bacillus* (0.13%), and others (1.71%), as indicated in Figure 3C. At the genus level, *Lactobacillus* and *Acetobacter* were the major bacteria present at approximately 73.36% and 21.06%, respectively, in Miang, followed by minor genera such as *Klebsiella* (1.01%), *Escherichia-Shigella* (0.68%), *Cohnella* (0.32%), *BIrii41* (0.31%), *Paenibacillus* (0.27%), *Limnochordaceae* 0.19%, *Bacillus* (0.13%), and others (2.72%) in Figure 3D.

The relative abundance of the bacterial communities at the genus level in Miang products fermented by the Luar ethnic group in Laos was compared with previous reports of microbial diversity in fermented tea products from Thailand and Myanmar, as represented in Table 1. Across various types of fermented tea products, there was a notable variation in microbial distribution in terms of abundance and composition. Specifically, the dominance of *Lactobacillus*, with over 79% relative abundance, was consistent with findings from Miang products in Thailand. For instance, FFP (filamentous fungi growth-based process) Miang with SSF (semi-submerged fermentation) exhibited a significant *Lactobacillus* composition of 82%, while FFP Miang with SMF (submerged fermentation) demonstrated a substantial presence of 77% [23]. This trend was also observed in NFP (non-filamentous fungi growth-based process) Miang [35], where *Lactobacillus* accounted for about 70%. On the other hand, a fermented tea product known as Laphet from Myanmar displayed a slightly lower frequency of *Lactobacillus*, ranging from 40% to 53%, while *Acetobacter* exhibited a notable presence in Laphet samples ranging [29] from 46% to 59%. In contrast to this, Miang fermented within bamboo tubes and NFP Miang exhibited lower levels of *Acetobacter*, around 21% to 22%. In particular, FFP Miang showed an absence of *Acetobacter*. Additionally, the detection of *Escherichia-Shigella* (0.68%) at the genus level was observed in a minor proportion. This could potentially be attributed to the presence of DNA remnants from dead cells of these microorganisms. Notably, coliform bacteria were not detected in Miang products due to the prolonged fermentation process of 3 to 5 weeks. This phenomenon may be attributed to the proposed antimicrobial activity linked to phenolic or phenolic-related compounds [19,33] and their potential antibacterial effect against pathogenic bacteria [18].

**Figure 4:**
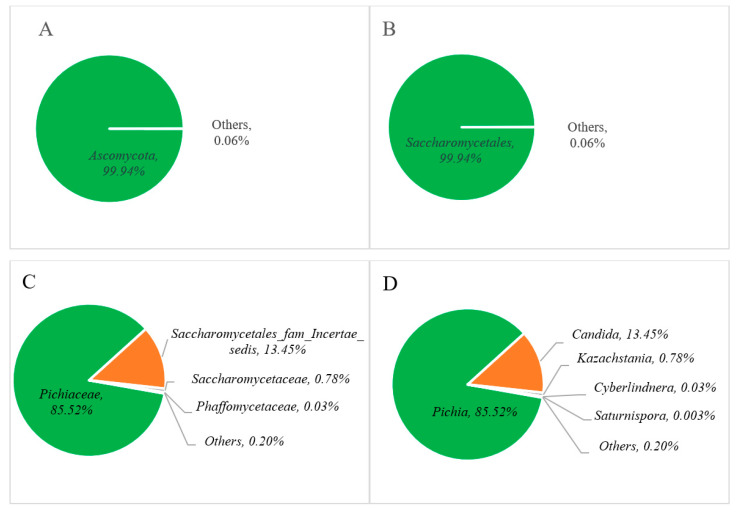
Relative abundance of the fungal communities at the phylum (**A**), order (**B**), family (**C**), and genus (**D**) levels of Miang.

The relative abundance of the fungal communities is represented in Figure 4, where phylum-level analysis reveals that *Ascomycota* dominates at over 99% (Figure 4A). At the class level, *Saccharomycetales* constitute the primary fungal community, with levels surpassing 99% (Figure 4B). Among these, the *Pichiaceae* family (85.52%) appeared as the predominant group (Figure 4C), followed by *Saccharomycetales fam Incertae sedis* (13.45%), *Saccharomycetaceae* (0.78%), *Phaffomycetaceae* (0.03%), and others (0.20%). At the genus level, *Pichia* (85.52%) and *Candida* (13.45%) were found as major fungal communities in Miang, followed by minor genera such as *Kazachstania* (0.78%), *Cyberlindnera* (0.03%), and others (2.72%) in Figure 4D.

The distribution of yeast diversity among various yeast taxa across different types of fermented tea products is presented in Table 2. In this study, yeast diversity at the genus level followed the order *Pichia* > *Candida* > *Kazachstania*. However, comparisons with previous research revealed differences in yeast diversity at the genus level. Kodchasee et al. [23] reported *Cyberlindnera* > *Pichia* > *Candida* in FFP Miang; in contrast, Unban et al. [32] reported that *Candida* > *Pichia* > *Cyberlindnera* were the major genera found in NFP Miang. Bo et al. [31] indicated that Laphet collected from tea plantation areas in Myanmar showed the following order of abundance: *Pichia* > *Debaryomyces* > *Cyberlindnera*. In contrast, Laphet collected from local markets in Myanmar showed *Dipodascus* > *Candida* > *Debaryomyces*. Remarkably, differences between samples sourced from tea plantations and local markets were significant. *Candida*, *Pichia*, and *Cyberlindnera* dominated in tea plantation samples, whereas *Debaryomyces* prevailed in local market samples [31]. These findings highlight the impact of geographical origin on microbial composition and the importance of the relationship between bacterial and yeast diversity during tea leaf fermentation, which arises from environmental conditions, processing methods, and microbial interactions [10]. The qualities of fermented tea, like other fermented foods, are formed by a combination of factors, such as the microbial community, regional characteristics, geographical conditions, and the specific fermentation process, which collectively determine the final product’s qualities [20,36].

### 3.3. pH and Total Acid Titration

**Figure 5:**
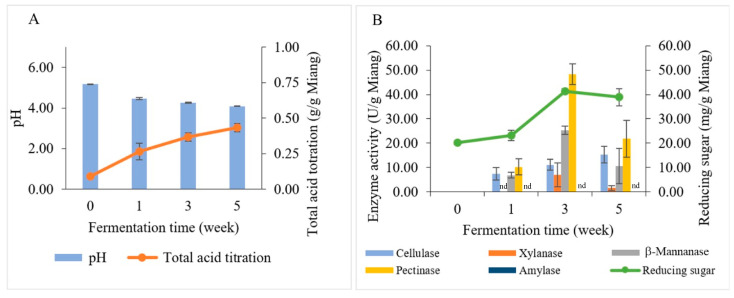
Changes in biochemical and polysaccharide-degrading enzymes during Miang fermentation: pH (bar chart) and total acid titration (linear line) (**A**). Enzyme activities (bar chart) and reducing sugar (linear line) (**B**). nd represents no enzyme activity was detected.

Changes in biochemical and polysaccharide-degrading enzymes during Miang fermentation are illustrated in Figure 5. The pH values and total acid titration of Miang are presented in Figure 5A. The initial pH value of steamed Miang (unfermented Miang) was 5.18; it dropped to 4.47 in the first week and then declined gradually to 4.26 and 4.09 at 3 weeks and 5 weeks, respectively. The total acid content of steamed Miang (unfermented) was 0.09 g/g Miang, and then through the fermentation process, total acid contents were increased constantly to 0.27, 0.37, and 0.43 g/g Miang, respectively. The increasing total acid contents were found similarly in prolonged fermentation in the Miang process [35], which can be typically explained by some carbohydrates being converted to organic acids, such as lactic acid and acetic acid, by microbial activity during fermentation [37].

### 3.4. Investigation of Polysaccharide-Degrading Enzymes in the Miang Fermentation Process

The reducing sugar and enzyme activities (β-mannanase, cellulase, pectinase, xylanase, and amylase) correlated to the Miang fermentation process were investigated by using the dinitrosalicylic acid (DNS) method (Figure 5B). The initial reducing sugar content was 20.18 mg/g Miang and then slightly increased to 23.13 mg/g Miang at week 1. Subsequently, it increased to the highest level of 41.29 mg/g Miang in week 3. After that, reducing sugar declined slightly to 38.89 mg/g Miang. The changes in reducing sugar during the Miang fermentation process might be the consequence of actions of polysaccharide hydrolysis enzymes caused by microbial growth that requires nutrition obtained from hydrolyzed polysaccharides [38]. The polysaccharide-degrading enzymes in the Miang fermentation process showed peaks of pectinase 48.32 U/g Miang, β-mannanase 25.32 U/g Miang, and xylanase 6.97 U/g Miang at week 3. Cellulase increased gradually and showed a peak of 15.33 U/g Miang in week 5; however, amylase was undetectable in Miang samples. The polysaccharide-degrading enzymes are expected to be produced by yeast due to their high number and capability to degrade polysaccharides. Furthermore, LAB have limited potential for polysaccharide degradation [39,40]. This trend was suggested in a previous study on traditional fermented tea, where pectinase was suggested to be produced by yeast [32]. Meanwhile, *Bacillus* spp. was suggested to produce β-mannanase based on a previous study that reported *Bacillus* spp. being isolated from Miang, which showed β-mannanase formation [22]. The polysaccharide degradation could be a valuable carbon source to derive and obtain energy and carbon sources for microbial succession [41,42,43]. Subsequently, the hydrolysis products obtained from enzyme activities were potentially utilized in metabolic pathways by microbial activity and transformed into organic acids, such as lactic and acetic acid.

### 3.5. Proximate Analysis

The nutritional components of Miang fermentation in bamboo tubes with underground incubation by the Luar ethnic group were examined through proximate analysis. The nutritional transformation was compared for each component during the Miang fermentation process for 5 weeks. The moisture content was 76.52% after steaming, and then dropped and remained constant at approximately 74.02–74.44% in the final products. This correlated with a change of nitrogen-free extract (NFE) and crude fiber, which was expected based on subsequent results from crude fiber degradation into NFE due to the complex carbohydrate being converted to usable nutrients by microbial cellulolytic enzymes for microbial growth [44]. The fat content showed a slight increase from initial levels of 1.3% to 2.52% in the final product, and this was an expected accumulation as part of microbial biomass in the fermentation process, where the fat component in tea leaves is suggested to be responding to create aroma and flavor characteristics [45,46]. On the other hand, protein content declined slightly from 17.21% to 16.05% after fermentation for 5 weeks, probably because protein content was metabolized by the microbes to be a nitrogen source for microbial growth [47,48]. The ash content was not significantly different at approximately 5.41–5.56%. A slight increase in ash content could be simply explained by reduced levels of other ratios, but structural components such as lignin or cellulose complex are not degraded, which potentially leads to higher ash content after fermentation.

### 3.6. Bioactive Compound Analysis

**Figure 6:**
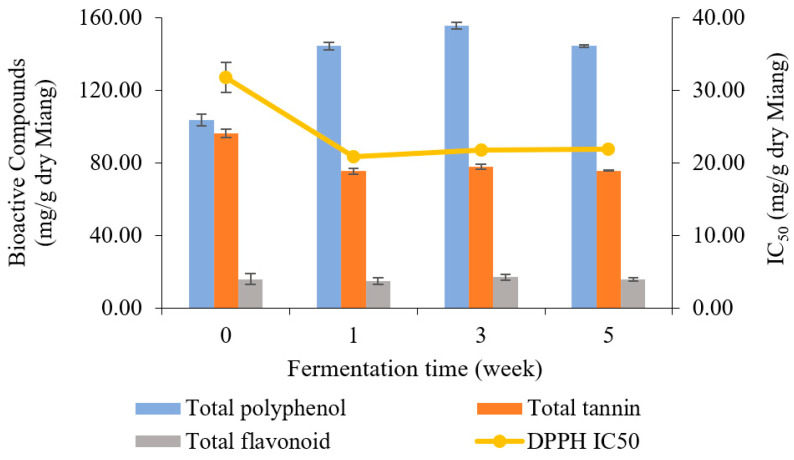
Changes in total polyphenol, total tannin, total flavonoid (bar charts), and antioxidant activity represented by IC_50_ (linear line) during Miang fermentation.

The bioactive compounds in Miang samples were analyzed periodically for 5 weeks. The results of TP, TA, TF, and antioxidant activity are presented in Figure 6. Overall, TP content was enhanced, while TA was decreased by fermentation. However, TF slightly fluctuated from the beginning to 5 weeks. The unfermented sample showed an initial total polyphenol content of 103.54 mg/g dry Miang, then fluctuated to 144.19, 155.52, and 144.91 mg/g dry Miang, respectively. Total tannin content dropped from the original level of 96.15 to 75.29 mg/g dry Miang in the first week, then held a steady trend until the end of fermentation. The changes in bioactive compounds were potentially induced by microbial biosynthetic and hydrolytic enzymes, which increased the extractability of polyphenols and complex forms and released new bioactive compounds [49,50,51,52]. Yeast isolates obtained from Miang fermentation could produce a tannin-degrading enzyme [17], which degraded tannin content, leading to reduced astringent or bitter taste in fermented tea [53,54,55]. Furthermore, biotransformation was involved in increasing total polyphenol derivatives in Miang fermentation, which may improve bioactive compounds such as antioxidant activity in terms of IC_50_ (half maximal inhibitory concentration) value. This was 31.74 μg/g dry Miang of the unfermented sample and decreased to 20.87, 21.75, and 21.81 μg/g dry Miang, respectively, in weeks 1, 3, and 5. This phenomenon was in agreement with previous studies [32], where Miang fermentation around 3–8 weeks is the appropriate fermentation time to get high antioxidant activity from the biotransformation.

### 3.7. Physical Properties of Color and Hardness

**Figure 7:**
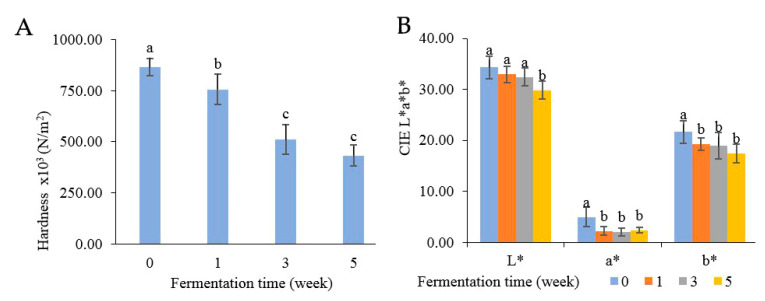
Changes in physical properties: hardness (**A**) and color (**B**) during Miang fermentation. The superscript letters indicate statistical significance (*p* < 0.05).

Texture analysis was measured in terms of hardness, as indicated in Figure 7A. At the beginning, the hardness of Miang was at 8.64 × 10^5^ N/m^2^ and then steadily declined to 4.32 × 10^5^ N/m^2^ until the end of week 5. The decreasing hardness after fermentation was potentially due to polysaccharide degradation from enzyme activities such as pectinase, β-mannanase, cellulase, and xylanase. The decreasing hardness was related directly to the quality of the final product [23,32]. The color changes are presented in Figure 7B, where the L* value represents the degree of lightness, the a* value indicates redness-green, and the b* value reflects yellow-blue. Overall, all attributes were decreased after fermentation. The lightness (L) gradually declined from 34.30 to 29.83 after 5 weeks. Redness (a*) and yellowness (b*) suddenly decreased after the first week and then remained steady (approximately 2.22–2.38 of redness and 19.26–17.44 of yellowness) until 5 weeks. The change in color might be caused by the oxidation of phenolic compounds such as theaflavins and thearubigins, which could transform some phenolic compounds into phenolic pigments, resulting in a natural browning reaction [56,57,58].

## 4. Conclusions

This research successfully investigated the microbial communities involved in the traditional Miang fermentation process within bamboo tubes using underground incubation, as practiced by the Luar ethnic group in Lao PDR. Among the identified microbes, *Lactobacillus* emerged as the predominant bacterial genus, closely followed by *Acetobacter*. *Pichia* exhibited the highest dominance in the fungal community, while *Candida* was present in smaller proportions. The fermentation process generated key polysaccharide-degrading enzymes, such as pectinase, β-mannanase, cellulase, and xylanase, generated through microbial activities. Notably, the microbial succession significantly influenced the final product’s physical and chemical attributes. The enzyme activities degraded tannins, reducing the astringent taste and contributing to a softer texture of the Miang, resulting in a more desirable product for consumption. The bioactive compounds in fermented Miang showed an increase in TP content during fermentation, indicating enhanced polyphenol extractability due to microbial activity. In contrast, the TA content dropped significantly due to the degradation of tannins by microbial enzymes. This microbial transformation process improved the bioactive compounds. Furthermore, a fermentation period of 3 weeks proved optimal for achieving increased antioxidant activity and bioactive compound content. Moreover, observing and allowing the appropriate fermentation duration can mitigate the risk of coliform bacterial presence, thus contributing to food safety and ensuring the successful Miang fermentation process within bamboo tubes through underground incubation.

## Figures and Tables

**Table 1:** Comparison of the bacterial communities’ relative abundance at the genus level in different types of final fermented tea products.

Taxonomy of Bacterial Diversity	ML Miang	NFP Miang	FFP Miang-SSF	FFP Miang-SMF	Laphet (PA)	Laphet (LM)
*Lactobacillus*	73.36%	70.65%	82.33%	77.05%	53.00%	40.00%
*Acetobacter*	21.02%	22.76%	-	-	46.00%	59.00%
*Klebsiella*	1.01%	2.29%	-	-	-	-
*Bacillus*	0.13%	0.07%	<0.01%	0.03%	-	-
*Plesiomonas*	-	1.70%	-	-	-	-
*Clostridium*	-	0.32%	-	-	-	-
*Cetobacterium*	-	0.63%	-	-	-	-
*Megashaera*	-	-	0.25%	1.01%	-	-
*Pantoea*	-	-	0.23%	0.19%	-	-
*Pluralibacter*	-	-	0.15%	0.17%	-	-
*Bacteroides*	-	-	0.06%	0.10%	-	-
*Bradyrhizobium*	-	-	0.01%	0.08%	-	-
*Sinorhizobium*	-	-	0.07%	0.05%	-	-
*Escherichia-Shigella*	0.68%	-	-	-	-	0.10%
*Acinetobacter*	-	-	<0.01%	0.03%	-	-
*Others*	3.80%	1.58%	17.11%	22.20%	1.00%	0.90%

ML: Miang prepared by the Luar ethnic group in Laos (as revealed in this research); NFP Miang: non-filamentous fungi growth-based process for 28 days in Thailand [32]; FFP Miang-SSF: filamentous fungi growth-based process in semi-submerged fermentation for 11 days, Thailand [23]; FFP Miang-SMF: filamentous fungi growth-based process in submerged fermentation at 11 days, Thailand [23]; Laphet (PA): Laphet product obtained randomly from a tea plantation area with a fermentation period ranging from 15 to 30 days in Ywarngan, Myanmar [31]; Laphet (LM): Laphet product obtained randomly from a local market with an unknown fermentation period in Pinlaung, Myanmar [31].

**Table 2:** Comparison of the fungal communities’ relative abundance at the genus level in different types of final fermented tea products.

Taxonomy of Fungal Diversity	ML	NFP Miang	FFP Miang-SSF	FFP Miang-SMF	Laphet (PA)	Laphet (LM)
*Pichia*	85.52%	8.77%	20.32%	2.56%	50.30%	16.00%
*Candida*	13.45%	82.91%	6.76%	4.39%	6.70%	20.00%
*Dipodascus*	-	-	-	-	32.00%	30.10%
*Debaryomyces*	-	0.34%	3.27%	1.17%	-	18.00%
*Cyberlindnera*	0.03%	2.00%	66.99%	87.53%	11.00%	14.00%
*Cryptococcus*	-	-	0.27%	0.11%	-	-
*Saccharomycetales*	-	-	-	-	-	1.80%
*Hanseniaspora*	0.03%	1.45%	-	-	<0.01%	-
*Kazachstania*	0.78%	-	-	-	<0.01%	-
*Wickerhamomyces*	-	0.08%	0.19%	0.06%	-	-
*Trichosporon*	-	-	0.02%	0.01%	-	-
*Mycosphaerella*	-	1.03%	-	-	-	-
*Others*	0.17%	3.41%	2.19%	4.18%	<0.01%	0.10%

ML: Miang prepared by the Luar ethnic group in Laos (as revealed in this research); NFP Miang: non-filamentous fungi growth-based process for 28 days in Thailand [32]; FFP Miang-SSF: filamentous fungi growth-based process in semi-submerged fermentation for 11 days, Thailand [23]; FFP Miang-SMF: filamentous fungi growth-based process in submerged fermentation at 11 days, Thailand [23]; Laphet (PA): Laphet product obtained randomly from a tea plantation area with a fermentation period ranging from 15 to 30 days in Ywarngan, Myanmar [31]; Laphet (LM): Laphet product obtained randomly from a local market with an unknown fermentation period in Pinlaung, Myanmar [31].

## Data Availability

The original contributions presented in the study are included in the article, further inquiries can be directed to the corresponding authors.
